# Design, Assessment, and *in vivo* Evaluation of a Computational Model Illustrating the Role of CAV1 in CD4^+^ T-lymphocytes

**DOI:** 10.3389/fimmu.2014.00599

**Published:** 2014-12-05

**Authors:** Brittany D. Conroy, Tyler A. Herek, Timothy D. Shew, Matthew Latner, Joshua J. Larson, Laura Allen, Paul H. Davis, Tomáš Helikar, Christine E. Cutucache

**Affiliations:** ^1^Department of Biology, University of Nebraska at Omaha, Omaha, NE, USA; ^2^Department of Biochemistry, University of Nebraska at Lincoln, Lincoln, NE, USA

**Keywords:** caveolin-1, CD4^+^ T-lymphocyte, the cell collective, adult T-cell leukemia, immunosuppression, immunotherapy, computational biology, logical models

## Abstract

Caveolin-1 (CAV1) is a vital scaffold protein heterogeneously expressed in both healthy and malignant tissue. We focus on the role of CAV1 when overexpressed in T-cell leukemia. Previously, we have shown that CAV1 is involved in cell-to-cell communication, cellular proliferation, and immune synapse formation; however, the molecular mechanisms have not been elucidated. We hypothesize that the role of CAV1 in immune synapse formation contributes to immune regulation during leukemic progression, thereby warranting studies of the role of CAV1 in CD4^+^ T-cells in relation to antigen-presenting cells. To address this need, we developed a computational model of a CD4^+^ immune effector T-cell to mimic cellular dynamics and molecular signaling under healthy and immunocompromised conditions (i.e., leukemic conditions). Using the Cell Collective computational modeling software, the CD4^+^ T-cell model was constructed and simulated under *CAV1*^+/+^, *CAV1*^+/−^, and *CAV1*^−/−^ conditions to produce a hypothetical immune response. This model allowed us to predict and examine the heterogeneous effects and mechanisms of CAV1 *in silico*. Experimental results indicate a signature of molecules involved in cellular proliferation, cell survival, and cytoskeletal rearrangement that were highly affected by *CAV1* knock out. With this comprehensive model of a CD4^+^ T-cell, we then validated *in vivo* protein expression levels. Based on this study, we modeled a CD4^+^ T-cell, manipulated gene expression in immunocompromised versus competent settings, validated these manipulations in an *in vivo* murine model, and corroborated acute T-cell leukemia gene expression profiles in human beings. Moreover, we can model an immunocompetent versus an immunocompromised microenvironment to better understand how signaling is regulated in patients with leukemia.

## Introduction

Caveolae are cave-like invaginations comprised mostly of the protein caveolin-1 (CAV1). In addition to the traditional roles of CAV1 in endocytosis, CAV1 has been implicated in processes ranging from signal transduction (1, 2), to both oncogenesis (3–5), and tumor suppression (6–8). Recently, three new roles for CAV1 emerged, including regulating immune synapse formation, T-cell receptor (TCR) activation, and mediating actin polymerization (9–11). Caveolin-1 knockout studies show an attenuated immune synapse formation as observed by decreased F-actin staining and dysregulation of RAC1 and ARP2/3 pathways (9). When T-cells engage with antigen-presenting cells (APCs), decreased TCR-dependent T-cell proliferation is observed when CAV1 is prohibited from interacting with CD26 (12). Downstream signaling pathways affected by CAV1 knockdown include the organization of the KSR1 mediated Raf/MEK/ERK signal cascade (13) and ZAP70, p56^lck^, and TCRζ phosphorylation (14). This mechanism has been shown to be distinct from CD3/CD28 stimulation (15), where no proliferation defects were observed in *Cav1*^−/−^ T-cells.

CAV1 acts as a scaffolding molecule thereby likely contributing to diverse events in the cell through CAV1-mediated recruitment of signaling complexes to the plasma membrane. Moreover, T-cell activation through the TCR and competent immune synapse formation are necessary for a healthy immune response. Misregulation of these processes can lead to deleterious effects, including cancer progression and a phenotype known as tumor-induced immunosuppression (16, 17). As CD4^+^ T-cells are vital for proper adaptive immune function, and CAV1 plays a role in immune synapse formation, we chose to further investigate the CAV1-mediated pathways in a CD4^+^ T-cell.

To better understand the intricate biology of CAV1 signaling in CD4^+^ T-cells, the development of a comprehensive *in silico* model is warranted. Through such a model, further identification of molecules associated with CAV1 signaling can occur.

Importantly, such a model allows for real-time simulations using computer software in an effort to identify specific mechanisms in the cell. In order to generate such a comprehensive and dynamic model, a systems biology approach is required (18). This approach provides a potentially greater understanding of the complex cellular functions that occur in living systems, allowing the use of computer models to conduct thousands of virtual experiments as well as make methodical predictions regarding proteins of interest (18–21).

Herein, we describe the construction and validation of a fully functional *in silico* CD4^+^ T-cell model using the Cell Collective, a web-based, open-source dynamic modeling platform that allows scientists to construct computational models in a non-mathematical fashion (20, 22). From this *in silico* starting position, comprehensive simulations were performed, allowing for predictions and hypotheses to be drawn for further *in vitro/in vivo* experimentation. To our knowledge, this is the first time a dynamic model of a CD4^+^ T-cell has been created to observe the downstream effects of *CAV1*^+/+^ (wild type), *CAV1*^+/−^ (heterozygous), and *CAV1*^−/−^ (knock down) upon cell signaling and intracellular networks as validated by *in silico* simulations and *in vivo* investigations.

## Materials and Methods

### Computational model construction with cell collective

The presented model was constructed using Cell Collective – a collaborative and interactive platform for modeling biological/biochemical systems (20, 22). The mathematical framework behind Cell Collective is based on a common qualitative (discrete) modeling technique where the regulatory mechanism of each node is described with a logical function [for more comprehensive information on logical modeling, see Ref. (23, 24)]. Cell Collective allows users to construct and simulate large-scale computational models of various biological processes based on qualitative interaction information extracted from previously published literature. The initial version of the model was structured after the previously published models (25, 26). The individual components and local interactions in the presented final model were retrieved manually from published literature. The model was subsequently validated against well-known experimentally demonstrated T-cell dynamics (see Model Validation), as well as new experiments presented in this paper. The Cell Collective’s Knowledge Base was used to catalog and annotate every interaction and regulatory mechanism (e.g., tyrosine phosphorylation on Y316) as mined from the primary literature. The model is freely available for simulations and further contributions by others directly in the platform. The model can be also downloaded in the SBML format (24) to be used within other software tools.

### Model validation

The model was constructed using *local* (e.g., protein–protein interaction) information from the primary literature. In other words, during the construction phase of the model, there was no attempt to determine the local interactions based on any other larger phenotypes or phenomena. However, after the model was completed, verification of the accuracy of the model involved testing it for the ability to reproduce complex input–output phenomena that have been observed in the laboratory. To do this, the T-cell model was simulated under a multitude of cellular conditions and analyzed in terms of input–output dose–response curves to determine whether the model behaves as expected [Figure 2; Ref. (27–33)], including various downstream effects as a result of activation of the TCR, G-protein-coupled receptor, cytokine, and integrin pathways. A total of 20 phenomena were used for the validation phase (data not shown).

### *In silico* simulations

The Cell Collective platform was utilized to perform all simulations for the CD4^+^ T-cell model. Virtual extracellular environments, composed of 20 CD4^+^ T-cell stimuli, were optimized for each *in silico* experiment based on immunocompetent versus immunocompromised (diseased) settings [Table 1; Ref. (9, 13, 30, 31, 34, 35)]. For each experiment, these values were analyzed and used to compare proteins most affected by *CAV1*^+/+^, *CAV1*^+/−^, and *CAV1*^−/−^ in an immunocompetent (i.e., WT) versus varying degrees of immunosuppression (i.e., Diseases A versus B) condition. The model was simulated under hypothetical disease-causing environments in order to observe the changes, if any, in CAV1 regulatory activity. Disease A mimics an immunosuppressive disease condition, and disease B simulates varying degrees of immunosuppression, as controlled by varying IL-10 levels. Each experiment consisted of 1,000 simulations, with different activity levels randomly selected between 0 and 100 for the external stimuli representing the extracellular environment. Each simulation consisted of 800 iterations and activity level of the model species was calculated over the last 300 iterations using methods previously described (36). The aforementioned simulations were run under six separate conditions including wild-type tissue, diseased tissue (diseases A and B), wild-type blood, and diseased blood (diseases A and B). Wild-type and diseased blood simulations are not included due to inconclusive data. Each experimental environment was simulated under (1) healthy cellular conditions, (2) CAV1 knocked out, (3) CAV1 activated 50% of the time (*CAV1*^+/−^) and (4) CAV1 activated at random levels between 0 and 100. (To be able to artificially control the activity levels of CAV1 under environments 3 and 4, an external species, “CAV1 Activator,” was built into the model to activate CAV1 independently of the activity levels of its direct upstream regulators).

### Mouse maintenance

Animals were housed in pathogen-free animal facilities, and all experimental protocols were reviewed and approved per the Institutional Animal Care and Use Committee at the University of Nebraska Medical Center/University of Nebraska at Omaha (IACUC# 13-056-08-EP). C57Bl/6J and B6.Cg-Cav1^tm1Mls^/J mice were purchased from the Jack-son Laboratory (Bar Harbor, ME, USA). Post-natal day 54 (±5 days) mice were used for all experiments.

### Histological staining

Spleen and lymph node tissues were sectioned and stained at the University of Nebraska Medical Center’s Tissue Science Facility. Spleen and lymph node tissues were sectioned, preserved in 10% formalin, and embedded onto slides in paraffin. Specifically, all tissues were sectioned after at least 48 h in fixative and stained with hematoxylin and eosin using standard protocol.

For immunohistochemical staining (IHC), tissues were deparrafinized in xylene for 3 min and rehydrated in decreasing concentrations of ethanol (100–50%). Antigen retrieval was performed by boiling sections in a solution of Sodium Citrate with 0.05% Tween-20. Blocking against non-specific binding and endogenous peroxidases was performed by incubation with 5% bovine serum albumin (BSA; Invitrogen) and 0.3% hydrogen peroxide, respectively. Primary antibody incubation was conducted for 90 min at room temperature in phosphate buffered saline (PBS; DIBCO). The following antibodies were used: Rac1 (Merck KGaA, Darmstadt, Germany); CD28 (BD Pharmingen); GATA3 (BD Pharmingen); CD26 (Abcam); BCL10 (Cell Applications, Inc.). Horse radish peroxidase-conjugated secondary antibodies (Cell Signaling Technology; BD Pharmingen; Abcam) were incubated for 1 h at room temperature. Slides were developed with a working solution 3,3-diaminobenzidine for 10 min at room temperature, followed by rinsing with distilled water and mounting the coverslip with Permount (Thermo Fisher Scientific).

### Gene expression profiling

Microarray data were downloaded from the Gene Expression Omnibus, accession number GSE55851 (37). Data contained whole genome expression profiling of CD4^+^ T-cells, sorted based on a CADM1/CD7 phenotype. Samples were collected from patients diagnosed with Adult T-cell leukemia-lymphoma (ATL) subtypes: asymptomatic (*n* = 2), smoldering (*n* = 2), chronic (*n* = 1), acute (*n* = 2), and healthy controls (*n* = 3). A minimum of two samples were taken from each patient for microarray analyses. Molecules of interest, as established utilizing the Cell Collective, were selected within the microarray data and analyzed by fold-change from normal controls between ATL subtypes. Fold-change values were subjected to uncentered, average-linkage correlation using Cluster 3.0, and Java TreeView as described previously (9). Further, Pearson regression analyses were conducted to discern correlation among molecules of interest in relation to CAV1 expression across ATL subtypes.

## Results

### *In silico* modeling of a CD4^+^ T-cell

**Figure 1:**
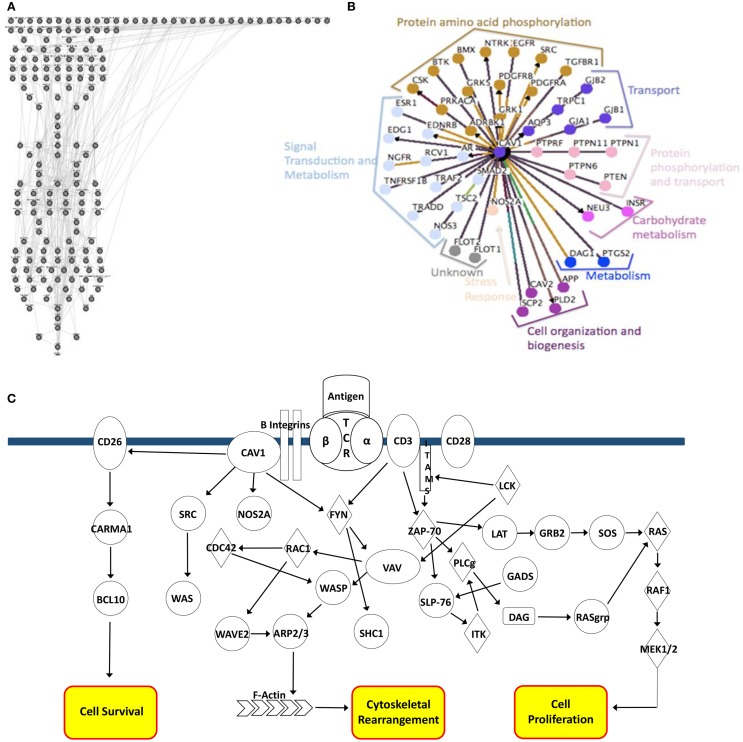
***In silico* modeling of a CD4^+^ T-cell**. **(A)** Nodal representation of CD4^+^ T-cell signaling pathways constructed using the Bio-Logic builder inclusive within the Cell Collective. Linkages represent protein–protein, protein–phosphorylation, and kinase interactions. **(B)** Osprey modeling of predicted CAV1 protein–protein interactions and functions. Linkages are categorized by function and centrality to CAV1. **(C)** Graphical depiction of CAV1-associated interactions. Major pathway end-points include cell survival, cytoskeletal rearrangement, and cellular proliferation.

The completed *CD4*^+^ *T-cell* model consists of 188 nodes representing components of various signaling pathways and corresponding protein-to-protein, protein-phosphorylation, and kinase interactions (Figure 1A). These interactions correlate with preliminary data generated using Osprey software used to model CAV1 protein-to-protein interaction types (Figure 1B). Each method of analysis (both the Cell Collective and Osprey) implicates a role for CAV1 in phosphorylation, signal transduction, transport, and cytoskeletal arrangement. The regulation of these events by CAV1 is highly complex and dynamic, as illustrated in Figure 1C.

**Figure 2:**
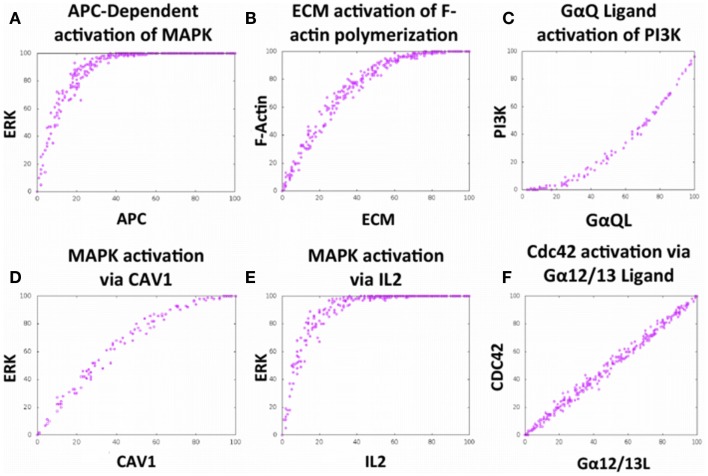
**The Cell Collective accurately models complex cellular phenomena**. **(A–F)** Certification of Bio-Logic built local interactions executing in accordance with primary literature findings. **(A)** Activation of the mitogen-activated protein kinase (MAPK) pathway via APC stimulation (27). **(B)** Positive relationship between filamentous actin polymerization in response to stimulation with extracellular matrix (ECM) components (28). **(C)** PI3-Kinase activation via binding of ligand to G protein-coupled receptor, GαQ (29). **(D)** Activation of the MAPK pathway via integrin-dependent ECM stimulation (9, 30). **(E)** Activation of the MAPK pathway via stimulation with interleukin-2 (IL2) (31, 32). **(F)** Activation of the small GTPase Cdc42 via binding of ligand to the G protein-coupled receptor, Gα12/13 (33); these results not shown in the graphic. Each dose–response curves appears to demonstrate a positive correlation with the stimulus.

To verify that the local interactions built into the model were able to accurately mimic complex phenomena that have been produced in the laboratory, 20 validations were conducted via simulations in Cell Collective (six representative validations are shown in Figures 2A–F). As an example, simulated TCR activation by an APC leads to Erk activation, and the subsequent downstream effect of cellular proliferation as represented by the literature (Figure 2A, Figure S1 in Supplemental Material).

### Identification of most affected proteins *in silico*

**Figure 3:**
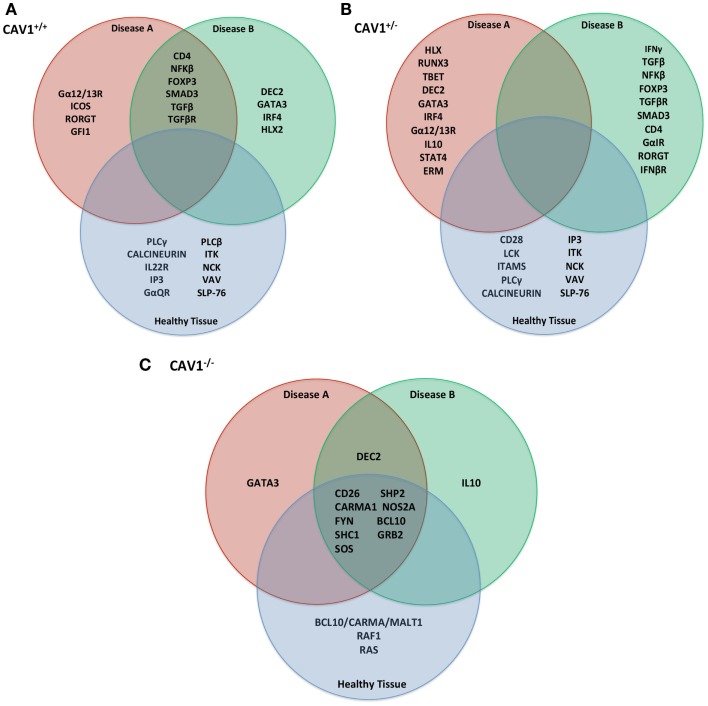
***In silico* predictions for translation into *in vitro/in vivo* experimentation**. Following 1,000 iterations of simulation as described in Table 1, the most affected proteins (either up or downregulated) were compiled by the Cell Collective and ranked based on activity% ON. Specifically, those described were the top 15 most differentially expressed molecules in the **(A)** *CAV1*^+/+^, **(B)** *Cav1*^+/−^, **(C)** *CAV1*^−/−^ genotype. These proteins were selected for further investigation with *in vitro/in vivo* verification.

Experiments were simulated using the *in silico* model (as described in Table 1) to make rational predictions about how the system would function in the laboratory. Following 1,000 iterations, we observed the protein products most affected by *CAV1*^+/+^, *CAV1*^+/−^, and *CAV1*^−/−^ in immounocompetent versus immunocompromised conditions (Figures 3A–C). In order to test the validity of the model, we chose to investigate expression levels of the proteins most affected in the knock down genotype as compared to a wild-type system. Specifically proteins disregulated by *CAV1*^−/−^ across all three conditions (WT, Disease A, and Disease B) included CD26, CARMA1, FYN, SHC1, SOS, SHP2, NOS2A, BCL10, and GRB2 (Figure 3C). Based on findings from the model as well as preliminary *in vitro* data, we hypothesized that CAV1 expression regulates Ras-related C3 botulinum toxin substrate 1 (RAC1), B-cell lymphoma/leukemia 10 (BCL10), GATA-binding protein 3 (GATA3), CD26, and CD28 (Figures 1C and 3C).

**Table 1:** **A summary of the experimental conditions simulated**.

External stimulus	Tissue (WT)	Tissue (Disease A)	Tissue (Disease B)
Alpha_13L	Med	High	High
GalphaS_L	Med	High	High
APC	Med	High	High
CGC	Med	Med-High	Med-High
ECM	High	High	High
GP130	0	0	0
IFNB	Med	Med	Med
IFNG	Med	Med	Med
IFNGR1	Med	Med	Med
IFNGR2	Med	Med	Med
IL10	Med	High	0–100
IL10RA	Med	High	High
IL10RB	Med	Med	Med
IL12	Med	High	High
IL15	Med	High	High
IL15RA	Med	High	High
IL18	Med	High	High
IL21	Med	High	High
IL22	Med	High	High
IL23	Med	High	High
IL27	Med	High	High
IL27RA	Med	Med	Med
IL2	Med	High	High
IL2RB	Med	High	High
IL4	Low	Low	Low
IL6	Low	Low	Low
IL6RA	Low	Low	Low
IL9	Low	Low	Low
TGFB	Low	Low	Low

*Specifically, these conditions included (1) a wild-type condition (i.e., healthy biological levels of cytokines), (2) an immunosuppressive disease condition (i.e., Disease A), and (3) a scenario with varying degrees of immunosuppression (i.e., Disease B) with *CAV1*^+/+^, *CAV1*^+/−^, and *CAV1*^−/−^*.

*Low, 0–20 activity level; Med, 21–60 activity level; High, 61–100 activity level (Note that the activity levels do not directly correspond to concentrations, rather the activity levels provide a semi-quantitative measure to describe the relative activity of a particular component of the model.)*.

The abovementioned results are indicative of CAV1-mediate regulation of a variety of cellular functions, notably those that are downstream of TCR and integrin pathways of which CAV1 serves as the scaffold. Given these results, we were able to make predictions about protein expression in relation to CAV1 and test them in the laboratory using *in vitro* and *in vivo* experiments. For example, we observed that CAV1 is involved in the integrin signaling pathway that ultimately activates the mitogen-activated protein kinase (MAPK) cascade (Figure 2); therefore, we can predict that cellular proliferation will be decreased if CAV1 is knocked down.

### Verification of in silico predictions *in vivo*

To determine differences in morphology between *CAV1*^−/−^ and wild-type mice, we examined tissues (including lymph nodes and spleen) stained with hematoxylin and eosin and observed no differences were observed in tissue architecture. Lymphoid organs (lymph nodes and spleen) were selected for their robustness of CD4^+^ T-cells, and liver was used as a control for tissue histology (i.e., to ensure mice were disease free). Furthermore, immunohistochemistry was utilized to biologically validate *in silico* predictions from our CD4^+^ T-cell model.

**Figure 4:**
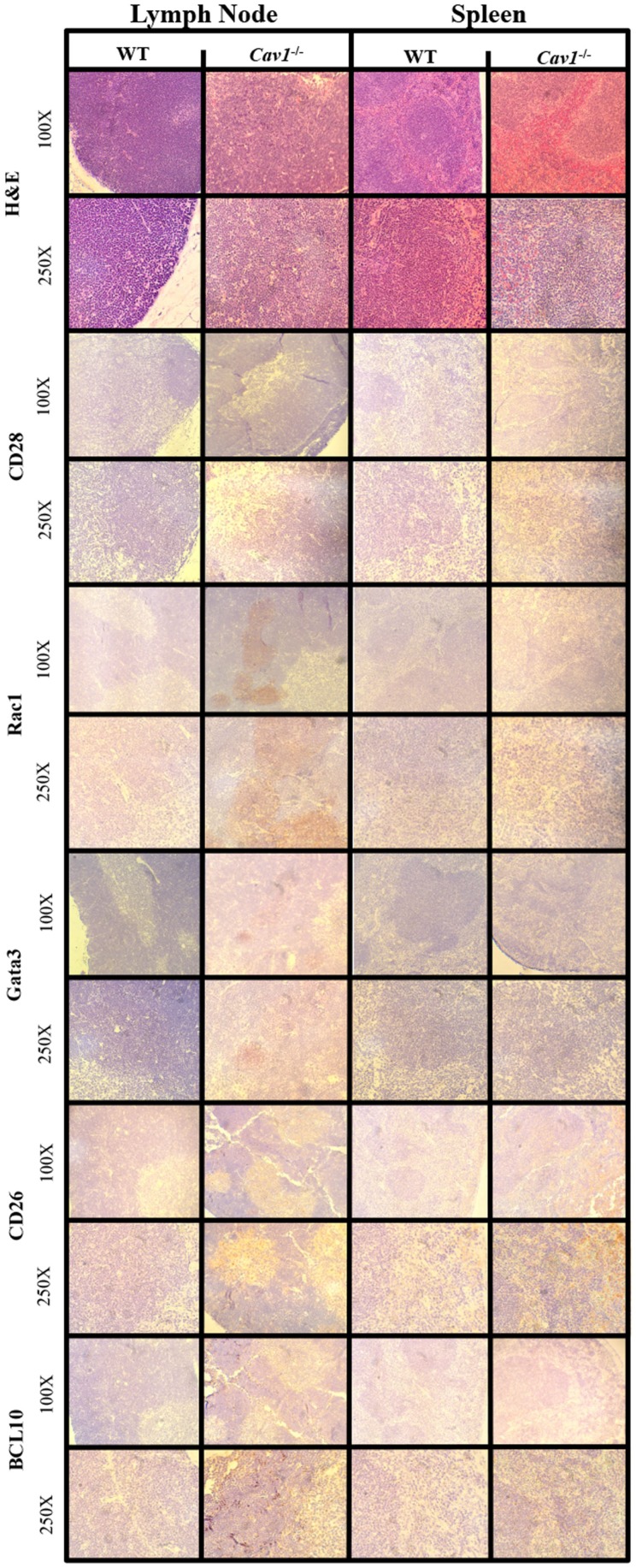
**Immunohistochemistry from murine model validation of *in silico-*predicted differentially expressed molecules with CAV1 knockout**. Molecules downstream of CAV1 that were predicted to be affected with CAV1 knockout using the Cell Collective were validated using lymphoid tissue histology from wild-type (WT) C57Bl/6 mice and *CAV1*^−/−^ mice. The corresponding hematoxylin and eosin preparations are included in the top panel for morphological orientation; all tissues were sectioned subsequently.

Top hit proteins: GATA3, RAC1, CD26, and BCL10 were selected for IHC to validate the model (Figure 4). RAC1 and GATA3 showed upregulation in the lymph node tissue of the *CAV1*^−/−^ mice. We observed CD26 and BCL10 to be upregulated in both the lymph node and spleen tissues of the *CAV1*^−/−^ mice. We also stained for CD28, as it is a well-characterized co-stimulatory protein involved in T-cell activation. CD28 showed no differential expression between the wild-type and *CAV1*^−/−^ mice.

**Figure 5:**
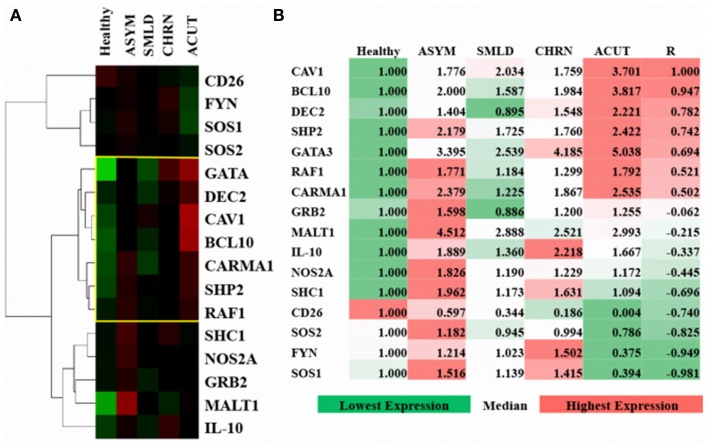
**Verification and comparison of *in silico* results with gene expression profiling from adult T-cell leukemia-lymphoma (ATL)**. **(A)** Uncentered average-linkage correlation of fold-change values from top affected proteins in ATL patients. The yellow-boxed region represents a CAV1-associated molecular signature (*R* = 0.78). Healthy (*n* = 3), asymptomatic (ASYM) (*n* = 2), smoldering (SMLD) (*n* = 2), chronic (CHRN) (*n* = 1), and acute (ACUT) (*n* = 2) cases are shown. A minimum of two samples were taken from each patient for microarray analyses. **(B)** Pearson regression analyses of top affected proteins in relation to CAV1 expression across ATL subtypes.

Based on predictions from the model (Figure 3C), we investigated the differential expression, hierarchal clustering (Figure 5A), and regression analyses (Figure 5B) of top hit proteins using microarray data from ATL cases (GSE55851). Comparison of ATL subtypes for identification of potential molecular signatures in relation to CAV1 expression reveal seven molecules, including CAV1, clustered together based upon gene expression profiles following hierarchal clustering (Figure 5A). We observed a positive correlation (*R* = 0.78), with distinct signatures displayed between healthy, asymptomatic, smoldering, chronic, and acute patients (Figure 5B). Pearson regression analyses were conducted between each molecule of interest in relation to CAV1 expression across ATL subtypes (Figure 5B). Specifically, we observed strong correlations between CAV1 and the following molecules: BCL10 (*R* = 0.947), DEC2/BHLHB3 (*R* = 0.782), SHP2/PTPN11 (*R* = 0.742), and GATA3 (*R* = 0.694). Conversely, SOS1 (*R* = −0.981), FYN (*R* = −0.949), SOS2 (*R* = -0.825), and CD26 (*R* = −0.740) were most negatively correlated to CAV1 expression. These data corroborate those described and simulated in the model (Figure 3); therefore, it is translated to *in vivo*, leukemic conditions.

## Discussion

Herein, we present a comprehensive, computational model of a CD4^+^ T-cell, including CAV1 regulatory pathways. This model incorporates experimentally validated interactions to posit the role of CAV1 in healthy CD4^+^ cells and CD4^+^ cells in the context of T-cell leukemia/lymphoma (i.e., when the immune response is skewed). CD4^+^ T-cells are a vital component of the immune system, as they protect against cancer, infection, and play a role in autoimmunity. CAV1 has been shown to be upregulated in numerous types of malignancies. Consequently, we built a CD4^+^ T-cell model to better understand basic T-cell biology and to address the role(s) of CAV1 within immunocompetent versus immunocompromised conditions. Inclusively, we investigated the role of CAV1 in the regulation of cellular processes, including cell cycle progression, cell proliferation, actin polymerization, and immune synapse formation. This model was successfully constructed using the Cell Collective platform that allows users to build cellular models capable of mimicking actual cellular systems in the laboratory (19–22, 38). The accuracy of the model was then successfully validated through the comparison of simulations with well-established, global input–output relationships as previously observed experimentally. Most importantly, new *in silico* predictions were validated *in vitro/in vivo* using both murine models and gene expression profiles from patients with a T-cell leukemia due to the previously observed role of Cav1 in lymphocytes (39–46).

Using Cell Collective, we were able to perform virtual experiments in order to make predictions as to how a CD4^+^ T-cell would behave when the expression levels of CAV1 were altered and when CAV1 was knocked down. These experiments provided insight as to which proteins, and ultimately which cellular functions, might be regulated by CAV1. Based on *in silico* results, we observed that BCL10, CD26, FYN, CARMA1, SHC1, SOS, SHP2, NOS2A, GRB2, and GATA3 are strongly influenced by CAV1 expression (Figure 3C). Consequently, the expression of these molecules *in vivo* was investigated using cluster analyses of microarray data to measure gene expression (Figure 5). Finally, four key proteins were selected for further verification of the predictions of the model using immunohistochemistry on mouse tissue with and without *Cav1* (Figure 4).

In addition to the four key proteins, CD28 was chosen because it is known to regulate T-cell activation independent of CAV1 expression (15). RAC1 and GATA3 expression were upregulated in the lymph node tissue of the *CAV1*^−/−^ mice. These results were expected based on studies showing the role of CAV1 in lymphoid tissue (9). We observed both CD26 and BCL10 upregulation, especially in the germinal centers of the lymph node and spleen tissue of the *CAV1*^−/−^ mice. These validations of the *in silico* predictions show that the CD4^+^ T-cell model is biologically relevant. These validations of the *in silico* predictions show that the CD4^+^ T-cell model is biologically relevant. Therefore, we suggest that based on the validations *in vivo* the T-cell *in silico* model is predictive of biological processes.

We then translated the *in silico* predictions to gene expression profiles of patients with a T-cell malignancy (i.e., ATL). Of the top 15 most differentially expressed molecules in CAV1-mediated pathways, the top 4 most highly correlated molecules to CAV1 expression were BCL10, DEC2, SHP2, and GATA3 (*R* value > 0.74; Figure 5B). Obstinately, SOS1, FYN, SOS2, and CD26 were negatively correlated with CAV1 (*R* < 0.74; Figure 5B). Additionally, we observed unique clustering of these 15 conserved molecules across subtypes of ATL patients. Interestingly, there was a distinguishable differential expression of MALT1 in asymptomatic ATL cases as compared with healthy individuals (Figure 5A). Therefore, further investigation into the plausibility of these correlated molecules being used for diagnostic purposes is warranted. We hope that our other ongoing studies will shed light as to their role in ATL. In short, our data regarding the role of CAV1 in cell signaling (as demonstrated using an *in silico* software and subsequently validated experimentally) corroborate that of the existing literature.

Specifically, CAV1 participates in processes including actin polymerization, cell proliferation, and cell survival (9, 12, 15, 47–49).

The usefulness of the *in silico* approach combined with that of *in vivo/in vitro* approaches provide rapid information as to the cell signaling networks in healthy and leukemic cells. There are currently many therapies being used to treat leukemia that target specific proteins in order to inhibit cellular pathways. These treatment modalities are advanced when comprehensive, molecular models allow the researcher to observe the direct mechanism of action of gene targeting as well as downstream consequences of gene/protein knockdown. The CD4^+^ T-cell model will hopefully be able to provide insight to for both T-cell biology (as demonstrated herein) as well as possible targets for lymphocytic leukemia treatments. The importance of *in silico* approaches combined with immunoinformatics as well as *in vivo* validation cannot be understated (18). With the often-prohibitive cost of drug design, it is imperative to use computational approaches to derive and test hypotheses. Current therapy regimens for T-cell malignancies can be modeled *in silico* initially in an effort to understand mechanisms and potential outcomes.

A comprehensive model of the CD4^+^ cells has the potential to provide substantial insight into cancer treatment, immunotherapy, and cellular biology. This twofold approach incorporating *in silico* and *in vivo* investigations has the potential to translate diagnostics and therapeutic targets from bench to bedside.

## Author Contributions

Brittany D. Conroy, Tyler A. Herek, and Timothy D. Shew are equally contributing first authors. Brittany D. Conroy wrote the paper, built the model, and did the simulations and verifications. Timothy D. Shew and Tyler A. Herek did bioinformatics studies, immunohistochemistry, verifications, and wrote the paper. Joshua J. Larson assisted in bioinformatics studies and mouse work. Laura Allen and Matthew Latner built the model, ran validations, and performed data analyses. Paul H. Davis provided suggestions for experimental design and helped implement experiments. Christine E. Cutucache and Tomáš Helikar conceptualized and designed the experiment, assisted in implementation, oversaw evaluation, and wrote the paper.

## Conflict of Interest Statement

Tomáš Helikar is a founder and scientific advisor to Discovery Collective, Inc. Discovery Collective holds a license to use the Cell Collective software. All other authors declare no conflict of interest.

## Supplementary Material

The Supplementary Material for this article can be found online at http://www.frontiersin.org/Journal/10.3389/fimmu.2014.00599/abstract
