# Supplementary figures and images for: Design, Assessment, and in vivo Evaluation of a Computational Model Illustrating the Role of CAV1 in CD4+ T-lymphocytes
Source: Front Immunol. 2014 Dec 5;5:599. doi: 10.3389/fimmu.2014.00599 (PMC4257089; doi:10.3389/fimmu.2014.00599)

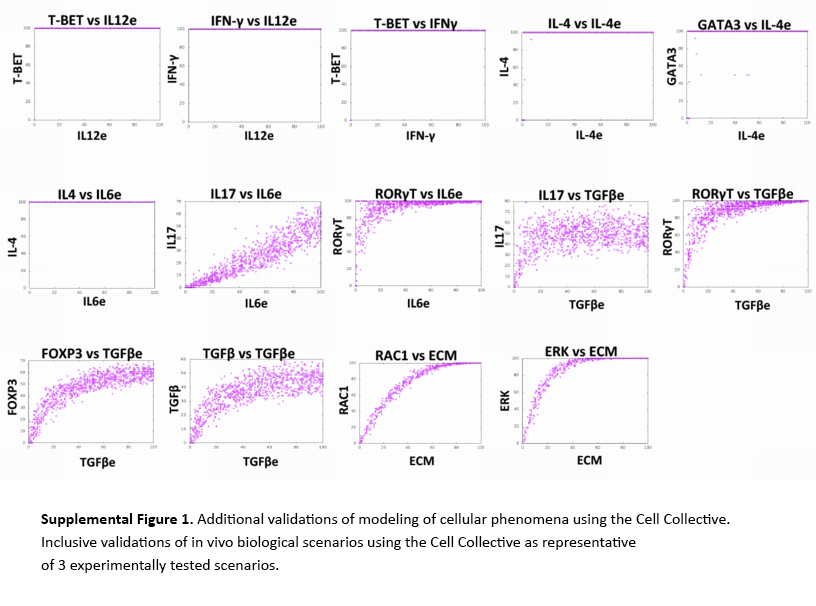

Supplement: Supplementary file 1 [file Image1.TIF]
